# The role of obesity, type 2 diabetes, and metabolic factors in gout: A Mendelian randomization study

**DOI:** 10.3389/fendo.2022.917056

**Published:** 2022-08-05

**Authors:** Yang Yang, Wei Xian, Dide Wu, Zijun Huo, Shubin Hong, Yanbing Li, Haipeng Xiao

**Affiliations:** Department of Endocrinology, The First Affiliated Hospital, Sun Yat-sen University, Guangzhou, China

**Keywords:** gout, urate, Mendelian randomization, causal relationship, metabolic factors

## Abstract

**Background:**

Several epidemiological studies have reported a possible correlation between risk of gout and metabolic disorders including type 2 diabetes, insulin resistance, obesity, dyslipidemia, and hypertension. However, it is unclear if this association is causal.

**Methods:**

We used Mendelian randomization (MR) to evaluate the causal relation between metabolic conditions and gout or serum urate concentration by inverse-variance-weighted (conventional) and weighted median methods. Furthermore, MR-Egger regression and MR-pleiotropy residual sum and outlier (PRESSO) method were used to explore pleiotropy. Genetic instruments for metabolic disorders and outcome (gout and serum urate) were obtained from several genome-wide association studies on individuals of mainly European ancestry.

**Results:**

Conventional MR analysis showed a robust causal association of increasing obesity measured by body mass index (BMI), high-density lipoprotein cholesterol (HDL), and systolic blood pressure (SBP) with risk of gout. A causal relationship between fasting insulin, BMI, HDL, triglycerides (TG), SBP, alanine aminotransferase (ALT), and serum urate was also observed. These results were consistent in weighted median method and MR-PRESSO after removing outliers identified. Our analysis also indicated that HDL and serum urate as well as gout have a bidirectional causal effect on each other.

**Conclusions:**

Our study suggested causal effects between glycemic traits, obesity, dyslipidemia, blood pressure, liver function, and serum urate as well as gout, which implies that metabolic factors contribute to the development of gout *via* serum urate, as well as potential benefit of sound management of increased serum urate in patients with obesity, dyslipidemia, hypertension, and liver dysfunction.

## Introduction

Gout is a disorder of purine metabolism which results from monosodium urate crystals in and around the joints caused by long-standing hyperuricemia (1). In developed countries, the prevalence of gout in men and women is 3%–6% and 1%–2%, respectively, which increases with age but stabilizes after the age of 70 (2). The concentration of uric acid is affected not only by environmental factors but also by inheritance. Several genome-wide association studies (GWAS) determined the relationship between SLC2A9, ABCG2, and SLC17A3 gene polymorphisms and uric acid concentration as well as gout (3, 4). Although the major cause of gout is well known, the understanding of its pathogenesis is still incomplete (5).

Several epidemiological studies have repeatedly indicated that gout is associated with obesity (6, 7). In addition, it has been reported that several metabolic diseases and factors including type 2 diabetes mellitus (T2DM), insulin resistance, dyslipidemia, and hypertension were associated with increased risk of gout or serum urate (8–10). Moreover, general obesity in women and hypertriglyceridemia in men may potentiate a hyperuricemia effect for gout development (11). Evidence indicated that hyperuricemia is associated with increased prevalence, incidence, and disease severity of non-alcoholic fatty liver disease (NAFLD), while NAFLD can predict hyperuricemia as well (12). Although these metabolic diseases and factors have been associated with gout or elevated serum urate in epidemiological studies, whether these associations are causal remains unclear because the associations may be confounded by an unhealthy diet or other behavioral or environmental risk factors.

To clarify the causal relationships between the observed associations, a Mendelian randomization is performed (13). There are three key assumptions for Mendelian randomization (MR) analysis: first, the genetic variants used as instrumental variables [single-nucleotide polymorphisms (SNPs)] should be robustly associated with the risk factor of interest (relevance assumption); second, the used genetic variants should not be associated with potential confounders (independent assumption); and third, the genetic variants should affect the risk of the outcome only through the risk factor, not *via* alternative pathways (exclusion restriction assumption) (14). In that way, MR evaluates the causal effect of an exposure on the outcome of interest by using genetic variants.

In the current study, we performed a two-sample Mendelian randomization analysis to investigate the causal relationship between metabolic exposures (including T2DM, obesity, blood lipid, blood pressure, and liver function) and serum urate or gout in individuals of mainly European ancestry. For this, summary level data from the GWAS on obesity, T2DM, metabolic factors, gout, and serum urate were included (13, 15–19).

## Materials and methods

### Study design and data sources

We investigate whether predisposition to metabolic traits [T2DM, fasting glucose, fasting insulin, body mass index (BMI), waist-to-hip ratio adjusted for body mass index (WHRadjBMI), blood lipid, blood pressure, and liver function] is likely to have an impact on gout and serum urate level, using MR.

A meta-analysis combining three GWAS data sets of European ancestry (62,892 T2DM and 596,424 controls) was used to identify genetic loci for T2DM (19). Results from the MAGIC (the Meta-Analyses of Glucose and Insulin-related traits Consortium) consortium were used to identify genetic proxies for glycemic traits (fasting glucose, fasting insulin) including 133,010 and 108,557 non-diabetic individuals of European ancestry, respectively (17). Summary statistics on BMI and WHRadjBMI were extracted from the Genetic Investigation of ANthropometric Traits consortium (GIANT) including 322,154 and 210,088 European ancestry individuals, respectively (15, 18).

Data (joint analysis of Metabochip and GWAS data) from the GWAS of Global Lipids Genetic Consortium (GLGC) were used to identify genetic loci for blood lipids [high-density lipoprotein cholesterol (HDL), low-density lipoprotein cholesterol (LDL), total cholesterol (TC), triglycerides (TG)] including 188,577 individuals mostly of European ancestry (20). Summary statistics for the association between SNP and blood pressure traits (systolic, diastolic, pulse pressure) were extracted from the largest genetic association study over one million people of European ancestry (13). Genetic instruments for liver function were identified from a recent genetic analysis in European-ancestry individuals (16).

The genetic data of outcomes (gout and serum urate) were derived from the Global Urate Genetics Consortium (GUGC). These data included 2,115 cases and 67,259 normal individuals from 14 European studies (21).

### Selection of instrumental variables

SNPs for each exposure trait were selected as instrumental variables (IV) according to the fundamental principle of MR. Each IV was independently [linkage disequilibrium (LD) r^2^ < 0.01] associated with the exposure traits at a genome-wide significance threshold (*P* < 5 × 10^-8^) in a previously published GWAS. Suitable proxy SNPs were chosen with the linkage disequilibrium (r^2^ > 0.8) to ensure that proxy SNP and target SNP have a strong correlation (22). Instrument strength in MR was evaluated with the F statistic derived from a measure of the exposure variance explained by each SNP. SNPs with instrument strengths (F) larger than 10 were selected (23). According to the principle mentioned above, we finally selected multiple independent SNPs strongly associated with each exposure trait and details of the included traits are displayed in **Table 1**.

**Table 1:** Related information of included traits in the Mendelian randomization analyses.

Trait	Variable type	Consortium	Ancestry
Type 2 diabetes	Exposure	eQTLGen	European
Fasting glucose	Exposure	MAGIC	European
Fasting insulin	Exposure	MAGIC	European
Body mass index	Exposure	GIANT	European
Waist-to-hip ratio	Exposure	GIANT	European
High-density cholesterol	Exposure	GLGC	Trans-ancestry
Low-density cholesterol	Exposure	GLGC	Trans-ancestry
Triglycerides	Exposure	GLGC	Trans-ancestry
Total cholesterol	Exposure	GLGC	Trans-ancestry
Systolic blood pressure	Exposure	ICBP	European
Diastolic blood pressure	Exposure	ICBP	European
Pulse pressure	Exposure	ICBP	European
Alkaline phosphatase	Exposure	UK Biobank	European
Alanine aminotransferase	Exposure	UK Biobank	European
Gout	Disease outcome	GUGC	European
Serum urate	Continuous outcome	GUGC	European

MAGIC, The Meta-Analyses of Glucose and Insulin-related traits Consortium; GIANT, Genetic Investigation of ANthropometric Traits consortium; ICBP, International Consortium for Blood Pressure; GLGC, Global Lipids Genetic Consortium; GUGU, Global Urate Genetics Consortium.

### Statistical analysis

In the main Mendelian randomization analyses, the inverse-variance-weighted (IVW) method was used to assess the causal associations (24). The Cochran Q test was used to assess heterogeneity between instrumental variables in the MR. We used random-effect models if the p value of the Cochran Q test was less than 0.05; otherwise, fixed-effect models were used (25). MR-Egger and the weighted median method were conducted to supplement the result of IVW. The MR-Egger method provided an estimate of horizontal pleiotropy from the intercept of a linear regression of SNP–outcome and SNP–exposure association estimate (26). In addition to the MR-Egger intercept, the MR-pleiotropy residual sum and outlier (PRESSO) method was also used to evaluate pleiotropy (27). Furthermore, MR-PRESSO is able to identify outlier variants based on their observed distance from the regression line and estimate results after correction of outliers (27). To control for false positive findings due to multiple testing, a conservative Bonferroni correction adjusted for the number of primary exposures analyzed in the study was applied, and *P*-values less than 0.003 were considered statistically significant. *P* values between 0.003 and 0.05 were deemed suggestive evidence of possible associations. Statistical analysis was conducted by R version 4.0.5. The R package “TwoSampleMR” version 0.5.6 and “MRPRESSO” were applied.

## Results

### Genetic association of type 2 diabetes and glycemic traits with gout and urate

**Figure 1:**
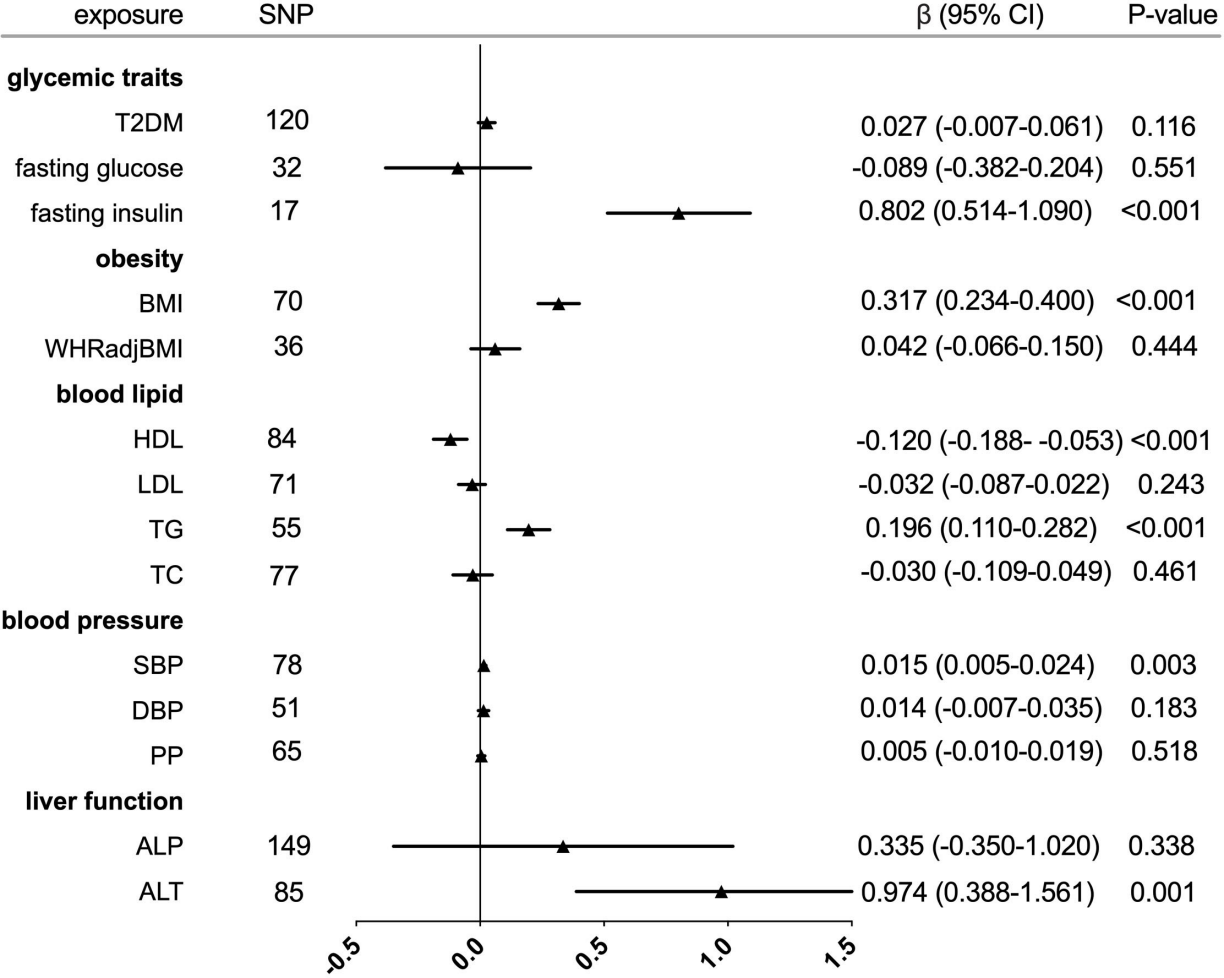
Forest plot of Mendelian randomization analyses for the genetical associations of glycemic traits, obesity, blood lipid, blood pressure, and liver function with increased serum urate. CI, confidence interval; SNP, single-nucleotide polymorphism.

**Figure 2:**
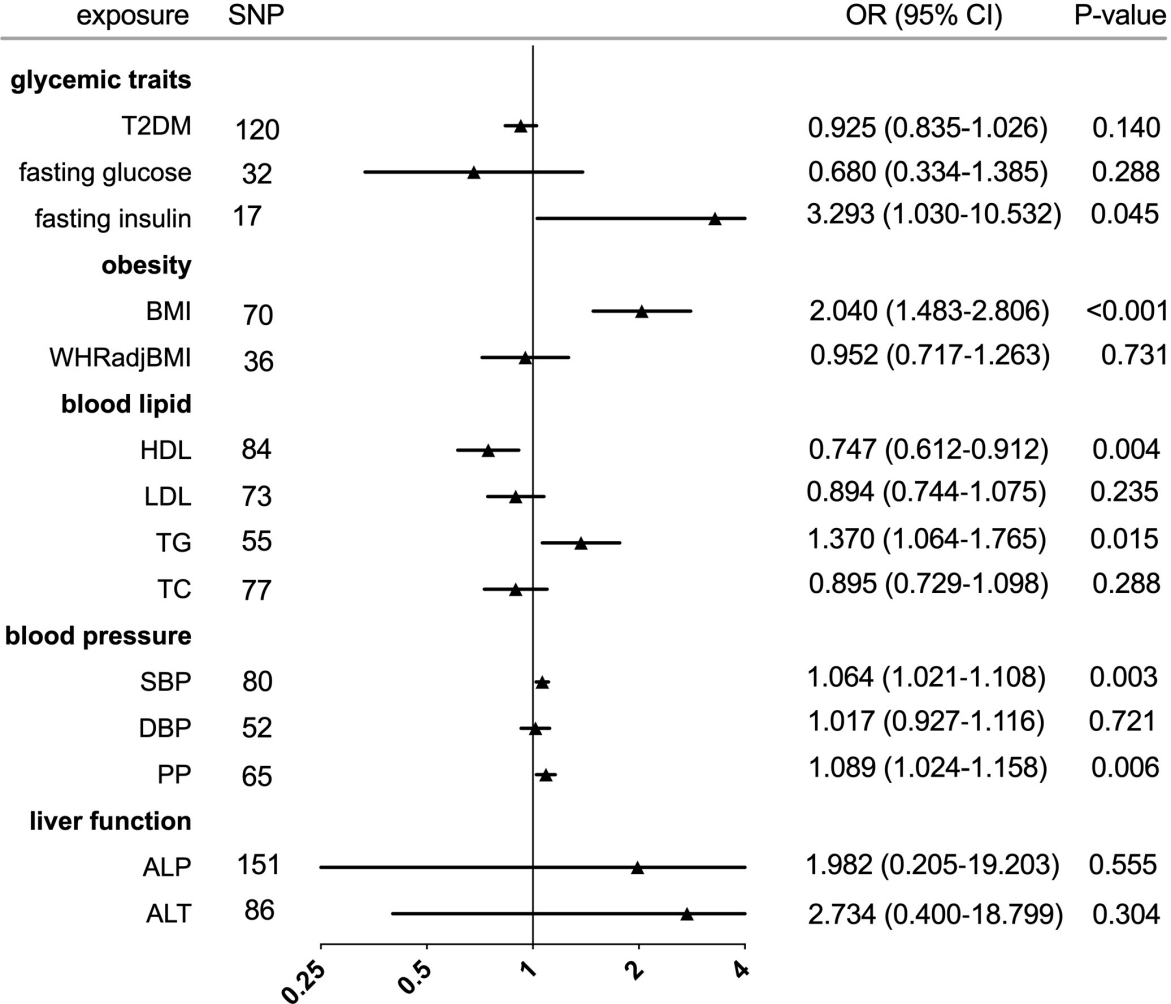
Forest plot of Mendelian randomization analyses for the genetical associations of glycemic traits, obesity, blood lipid, blood pressure, and liver function with risk of gout. CI, confidence interval; SNP, single-nucleotide polymorphism; OR, odds ratio.

The individual instrument-exposure (T2DM, fasting glucose, and fasting insulin) is shown in **Supplementary Table 1**. IVW MR using associated instrumental SNPs indicated that T2DM (β = 0.027, 95% CI: -0.007-0.061, *P* = 0.116) was not causally associated with serum urate level and gout, which was also supported by weighted median and MR-Egger analysis **(** **Figures 1**, **2** and **Supplementary Figure 1** **)**. However, post-removal of outliers identified by MR-PRESSO suggested a significance of the association between T2DM and increased serum urate (β = 0.652, 95% CI: 0.029–0.080, *P* < 0.001) **(** **Table 2** **)**.

**Table 2:** Mendelian randomization estimates of glycemic traits on serum urate or gout.

Outcome	Exposure	Method	Estimate	95% CI	P-value	MR-Egger intercept (P-value)
Serum urate	T2DM	IVW MR	0.027	-0.007-0.061	0.116	0.004 (0.112)
		Weighted median	0.029	-0.001-0.059	0.058	
		MR-Egger	-0.029	-0.104-0.046	0.456	
		MR-PRESSO	0.052	0.029-0.080	<0.001	
	FBG	IVW MR	-0.089	-0.382-0.204	0.551	0.005 (0.494)
		Weighted median	-0.028	-0.170-0.115	0.702	
		MR-Egger	-0.268	-0.854-0.318	0.377	
		MR-PRESSO	0.017	-0.116-0.15	0.802	
	FI	IVW MR	0.802	0.514-1.090	<0.001	0.005 (0.723)
		Weighted median	0.891	0.603-1.179	<0.001	
		MR-Egger	0.483	-1.272-2.237	0.597	
		MR-PRESSO	0.813	0.577-1.049	<0.001	
Gout	T2DM	IVW MR	0.925	0.835-1.026	0.140	0.002 (0.816)
		Weighted median	1.001	0.836-1.199	0.988	
		MR-Egger	0.903	0.719-1.135	0.383	
		MR-PRESSO	0.943	0.855-1.040	0.240	
	FBG	IVW MR	0.680	0.334-1.385	0.288	<0.001 (0.993)
		Weighted median	1.056	0.467-2.389	0.896	
		MR-Egger	0.676	0.159-2.870	0.599	
		MR-PRESSO	0.888	0.478-1.647	0.708	
	FI	IVW MR	3.293	1.030-10.532	0.045	-0.002 (0.968)
		Weighted median	4.106	0.952-17.705	0.058	
		MR-Egger	3.803	0.789-18.324	0.716	
		MR-PRESSO	3.293	1.030-10.532	0.062	

T2DM, type 2 diabetes; FBG, fasting glucose; FI, fasting insulin.

In the IVW analysis, fasting insulin-associated SNPs in Europeans showed no causal effect on increased serum urate and risk of gout (*P* = 0.287 and *P* = 0.635, respectively). After removing rs780094 in the GCKR gene according to leave-one-out analysis, recalculating the main IVW estimate suggested a causal effect of fasting insulin on increased serum urate (β = 0.802, 95% CI: 0.514–1.090, *P* < 0.001) and risk of gout (OR = 3.293, 95% CI: 1.030–10.532, *P* = 0.045) **(** **Figures 1**, **2** and **Supplementary Figure 2** **)**. In addition, weighted median analysis suggested a causal effect of fasting insulin on increased serum urate (β = 0.891, 95% CI: 0.603–1.179, *P* < 0.001). The MR estimate after removing outliers identified by MR-PRESSO showed similar results (β = 0.813, 95% CI: 0.577–1.049, *P* < 0.001) **(** **Table 2** **)**. To further investigate the relationship between serum urate and fasting insulin, we performed bidirectional MR analyses assessing the effects of genetically predicted serum urate on fasting insulin. We observed no causal effects of serum urate on fasting insulin (**Supplementary Table 2**).

The relationship between fasting glucose and serum urate was also investigated. IVW analysis revealed that fasting glucose was not associated with serum urate and gout **(** **Figures 1**, **2** and **Supplementary Figure 3** **)**. There was no evidence of horizontal pleiotropy influencing the estimates in that the MR-Egger intercept was not significant **(** **Table 2** **).** Leave-one-variant-out analysis was conducted to identify variants with exaggerated influence on the combined effect estimate **(** **Supplementary Table 3** **)**.

### Genetic association of obesity with gout and urate

The individual instrument-exposure (BMI and WHRadjBMI) is shown in **Supplementary Table 4**. Using the IVW analysis, BMI was associated with an increase in the level of serum urate (β = 0.317, 95% CI: 0.234–0.400, *P* < 0.001) and risk of gout (OR = 2.040, 95% CI:1.483–2.806, *P* < 0.001) based on all SNPs in Europeans, with similar and significant results for weighted median analysis **(** **Figures 1**, **2** and **Supplementary Figure 4** **)**. In the MR-Egger analysis, BMI was associated with an increase in serum urate level, with a similar but non-significant result in gout. MR-Egger did not show evidence of horizontal pleiotropy. The MR-PRESSO test showed pleiotropy in serum urate (*P* < 0.001), but not in gout (*P* = 0.874). The MR-PRESSO distortion test was significant for BMI on serum urate after removing outliers (β = 0.341, 95% CI: 0.266–0.416, *P* < 0.001) **(** **Table 3** **)**. Moreover, MR analyses about the effects of genetically predicted serum urate and gout on BMI were also conducted. IVW analysis revealed that serum urate and gout were not associated with BMI (**Supplementary Table 3**).

**Table 3:** Mendelian randomization estimates of obesity on serum urate or gout.

Outcome	Exposure	Method	Estimate	95% CI	P-value	MR-Egger intercept (P-value)
serum urate	BMI	IVW MR	0.317	0.234-0.400	<0.001	0.001 (0.805)
		weighted median	0.350	0.260-0.441	<0.001	
		MR-Egger	0.288	0.043-0.533	0.024	
		MR-PRESSO	0.341	0.266-0.416	<0.001	
					
	WHRadjBMI	IVW MR	0.042	-0.066-0.150	0.444	-0.002 (0.842)
		weighted median	0.026	-0.098-0.149	0.684	
		MR-Egger	0.097	-0.449-0.642	0.730	
		MR-PRESSO	0.042	-0.066-0.150	0.449	
						
gout	BMI	IVW MR	2.040	1.483-2.806	<0.001	0.003 (0.833)
		weighted median	2.076	1.295-3.328	0.002	
		MR-Egger	1.858	0.740-4.664	0.191	
		MR-PRESSO	2.039	1.521-2.734	<0.001	
					
	WHRadjBMI	IVW MR	0.952	0.717-1.263	0.731	-0.029 (0.184)
		weighted median	1.040	0.710-1.522	0.841	
		MR-Egger	2.257	0.628-8.112	0.221	
		MR-PRESSO	0.952	0.720-1.258	0.730	

BMI, Body mass index ; WHRadjBMI, Waist-to-hip ratio adjusted for body mass index.

IVW MR using 36 associated instrumental SNPs indicated that WHRadjBMI was not causally associated with serum urate level (β = 0.042, 95% CI: -0.066–0.150, *P* = 0.444) and gout (OR = 0.952, 95% CI: 0.717–1.263, *P* = 0.731) **(** **Figures 1**, **2** and **Supplementary Figure 5** **)**. As shown in **Table 3**, the MR-Egger intercepts did not provide evidence of horizontal pleiotropy in any analysis, and neither did MR-PRESSO identify outliers. For both serum urate and gout, leave-one-variant-out analysis did not identify variants with exaggerated influence on the combined effect estimate **(** **Supplementary Table 5** **)**.

### Genetic association of serum lipid with gout and urate

The individual instrument-exposure (HDL, LDL, TG, and TC) is shown in **Supplementary Table 6**. In the IVW analysis, HDL-associated SNPs had a causal effect on decreased serum urate (β = -0.012, 95% CI:-0.188–0.053, *P* < 0.001) and risk of gout (OR = 0.747, 95% CI: 0.612–0.912 *P* = 0.004) **(** **Figures 1**, **2** and **Supplementary Figure 6** **)**. The MR-Egger test precluded the possibility of horizontal pleiotropy of instrument variables. After Bonferroni correction, the result was deemed suggestive evidence of a possible association between HDL and risk of gout (0.003 < *P* < 0.05). To explain this association more accurately, weighted median and MR-PRESSO analyses were conducted. Although possible outlier SNPs were identified in serum urate using the MR-PRESSO test, the effect estimate of the association between genetically predicted HDL and serum urate did not change markedly after outlier correction (β = -0.139, 95% CI: -0.184–0.095, *P* < 0.001) **(** **Table 4** **)**. When setting HDL as the outcome, serum urate and gout were causally associated with HDL, as shown in **Supplementary Table 3**.

**Table 4:** Mendelian randomization estimates of blood lipid on serum urate or gout.

Outcome	Exposure	Method	Estimate	95% CI	P-value	MR-Egger intercept (P-value)
Serum urate	HDL	IVW MR	-0.120	-0.188- -0.053	<0.001	-0.007 (0.015)
		Weighted median	-0.094	-0.150- -0.038	<0.001	
		MR-Egger	0.013	-0.111-0.136	0.841	
		MR-PRESSO	-0.139	-0.184- -0.095	<0.001	
					
	LDL	IVW MR	-0.032	-0.087-0.022	0.243	-0.004 (0.125)
		Weighted median	-0.009	-0.055-0.036	0.691	
		MR-Egger	0.020	-0.065-0.105	0.654	
		MR-PRESSO	-0.012	-0.050-0.027	0.555	
					
	TG	IVW MR	0.196	0.110-0.282	<0.001	0.009 (0.376)
		Weighted median	0.095	0.031-0.159	0.024	
		MR-Egger	0.162	0.025-0.299	0.034	
		MR-PRESSO	0.127	0.067-0.186	<0.001	
					
	TC	IVW MR	-0.030	-0.109-0.049	0.461	-0.005 (0.176)
		Weighted median	-0.047	-0.103-0.010	0.106	
		MR-Egger	0.068	-0.093-0.228	0.411	
		MR-PRESSO	-0.035	0.081-0.012	0.149	
Gout	HDL	IVW MR	0.747	0.612-0.912	0.004	-0.013 (0.121)
		Weighted median	0.873	0.653-1.167	0.359	
		MR-Egger	0.960	0.662-1.391	0.829	
		MR-PRESSO	0.759	0.626-0.920	0.006	
					
	LDL	IVW MR	0.894	0.744-1.075	0.235	-0.003 (0.710)
		Weighted median	0.971	0.753-1.253	0.821	
		MR-Egger	0.934	0.697-1.249	0.645	
		MR-PRESSO	0.888	0.742-1.062	0.198	
					
	TG	IVW MR	1.370	1.064-1.765	0.015	0.009(0.376)
		Weighted median	1.006	0.723-1.401	0.971	
		MR-Egger	1.189	0.795-1.778	0.404	
		MR-PRESSO	1.370	1.064-1.765	0.015	
					
	TC	IVW MR	0.895	0.729-1.098	0.288	-0.006 (0.532)
		Weighted median	0.809	0.609-1.073	0.143	
		MR-Egger	1.007	0.661-1.522	0.975	
		MR-PRESSO	0.882	0.728-1.069	0.206	

HDL, high-density cholesterol; LDL, low-density cholesterol; TG, triglycerides; TC, total cholesterol.

Using 55 associated SNPs as instrumental variables, IVW MR analysis evaluated that the level of TG had a significant effect on increased serum urate (β = 0.196, 95% CI: 0.110–0.282, P < 0.001) **(** **Figures 1**, **2** and **Supplementary Figure 7** **)**. After Bonferroni correction, TG and risk of gout were not causally associated (*P* = 0.015). MR-Egger did not show evidence of horizontal pleiotropy. After removal of outliers, the magnitude and significance of the association between TG and serum urate remained in the MR-PRESSO analysis **(** **Table 4** **)**. When setting serum urate as the exposure, serum urate was causally associated with TG, as shown in **Supplementary Table 3**. Leave-one-variant-out analysis was performed to identify variants with exaggerated influence on the combined-effect estimate **(** **Supplementary Table 7** **)**. However, instruments for the other lipid traits, including LDL and TC, did not indicate an effect on serum urate and gout **(** **Figures 1**, **2** and **Supplementary Figure 8**, **9** **)**.

### Genetic association of blood pressure with gout and urate

The individual instrument-exposure [systolic blood pressure (SBP), diastolic blood pressure (DBP), pulse pressure (PP)] is shown in **Supplementary Table 8**. In the IVW analysis, SBP-associated SNPs in Europeans had a causal effect on increased serum urate (β = 0.015, 95% CI: 0.005–0.024, *P* = 0.003) and risk of gout (OR = 1.064, 95% CI: 1.021–1.108, *P* = 0.003), which was also supported by weighted median analysis **(** **Figures 1**, **2** and **Supplementary Figure 10** **)**. MR-Egger did not show evidence of horizontal pleiotropy. The MR-PRESSO test showed pleiotropy in serum urate (P < 0.001), but not in gout (*P* = 0.512). Although possible outlier SNPs were identified in serum urate using the MR-PRESSO test, the effect estimate of the association between genetically predicted SBP and serum urate did not change markedly after outlier correction (β = 0.017, 95% CI: 0.008–0.026, P < 0.001) (**Table 5**). When setting SBP as the outcome, genetically determined serum urate and gout were not associated with SBP **(** **Supplementary Table 3** **)**. IVW MR using 65 associated instrumental SNPs indicated that PP was causally associated with gout (OR = 1.089, 95% CI: 1.024–1.158, *P* = 0.006) and the weighted-median method also produced a similar result (OR = 1.100, 95% CI: 1.005–1.204, *P* = 0.039), which were not significant after Bonferroni correction **(** **Figures 1**, **2** and **Supplementary Figure 11** **)**. The MR-Egger intercepts did not provide evidence of horizontal pleiotropy, and neither did MR-PRESSO identify outliers. Instruments for the DBP did not indicate an effect on serum urate and gout **(** **Table 5** and **Supplementary Figure 12** **)**. Leave-one-variant-out analysis did not identify variants with exaggerated influence on the combined effect estimate **(** **Supplementary Table 9** **)**.

**Table 5:** Mendelian randomization estimates of blood pressure on serum urate or gout.

Outcome	Exposure	Method	Estimate	95% CI	P-value	MR-Egger intercept (P-value)
Serum urate	SBP	IVW MR	0.015	0.005-0.024	0.003	-0.002 (0.717)
		Weighted median	0.016	0.006-0.027	0.002	
		MR-Egger	0.024	-0.029-0.076	0.376	
		MR-PRESSO	0.017	0.008-0.026	<0.001	
					
	DBP	IVW MR	0.014	-0.007-0.035	0.183	0.001 (0.790)
		Weighted median	0.010	-0.011-0.030	0.365	
		MR-Egger	0.005	-0.068-0.077	0.896	
		MR-PRESSO	0.007	-0.011-0.024	0.450	
					
	PP	IVW MR	0.005	-0.010-0.019	0.518	-0.009 (0.076)
		Weighted median	0.002	-0.014-0.018	0.773	
		MR-Egger	0.061	-0.002-0.125	0.061	
		MR-PRESSO	0.011	-0.002-0.023	0.099	
Gout	SBP	IVW MR	1.064	1.021-1.108	0.003	0.007 (0.783)
		Weighted median	1.063	1.060-1.123	0.031	
		MR-Egger	1.031	0.821-1.294	0.793	
		MR-PRESSO	1.061	1.020-1.104	0.005	
					
	DBP	IVW MR	1.017	0.927-1.116	0.721	-0.001 (0.963)
		Weighted median	1.018	0.910-1.138	0.759	
		MR-Egger	1.025	0.743-1.413	0.883	
		MR-PRESSO	1.001	0.943-1.063	0.966	
					
	PP	IVW MR	1.089	1.024-1.158	0.006	-0.020 (0.334)
		Weighted median	1.100	1.005-1.204	0.039	
		MR-Egger	1.242	0.946-1.630	0.123	
		MR-PRESSO	1.091	1.032-1.153	0.003	

SBP, systolic blood pressure; DBP, diastolic blood pressure; PP, pulse pressure.

### Genetic association of liver function with gout and urate

The individual instrument-exposure [alkaline phosphatase (ALP) and alanine aminotransferase (ALT)] is shown in **Supplementary Table 10**. Using 85 associated SNPs as instrumental variables, IVW MR analysis evaluated that the level of ALT had a significant effect on increased serum urate (β = 0.974, 95% CI: 0.388–1.561, *P* = 0.001) in Europeans **(** **Figures 1**, **2** and **Supplementary Figure 13** **)**. The result was similar with that in weighted median analysis **(** **Table 6** **)**. The MR-Egger intercepts showed evidence of horizontal pleiotropy in serum urate (MR-Egger intercept = 0.009, *P* = 0.004). The MR estimate after removing six outliers identified by MR-PRESSO suggested a significance of the association between ALT and increased serum urate (β = 0.879, 95% CI: 0.355–1.404, *P* = 0.002) **(** **Table 6** **)**. Leave-one-variant-out analysis did not identify variants with exaggerated influence on the combined effect estimate **(** **Supplementary Table 11** **)**. To investigate the relationship between serum urate, gout, and fasting insulin, bidirectional MR analyses were conducted to indicate that genetically determined serum urate and gout were not associated with ALT **(** **Supplementary Table 3** **)**. Instruments for the ALP did not indicate an effect on serum urate and gout **(** **Figures 1**, **2** and **Supplementary Figure 14** **)**.

**Table 6:** Mendelian randomization estimates of liver function on serum urate or gout.

Outcome	Exposure	Method	Estimate	95% CI	P-value	MR-Egger intercept (P-value)
Serum urate	ALP	IVW MR	0.335	-0.350-1.020	0.338	0.002 (0.821)
		Weighted median	-0.088	-0.806-0.630	0.811	
		MR-Egger	0.147	-1.106-1.401	0.818	
		MR-PRESSO	0.304	-0.181-0.789	0.222	
					
	ALT	IVW MR	0.974	0.388-1.561	0.001	0.009 (0.004)
		Weighted median	0.627	0.042-1.211	0.036	
		MR-Egger	-0.757	-2.032-0.517	0.247	
		MR-PRESSO	0.879	0.355-1.404	0.002	
Gout	ALP	IVW MR	1.982	0.205-19.203	0.555	0.001 (0.727)
		Weighted median	2.917	0.081-105.098	0.558	
		MR-Egger	1.107	0.010-119.384	0.196	
		MR-PRESSO	1.985	0.249-15.807	0.835	
					
	ALT	IVW MR	2.743	0.400-18.799	0.304	0.001 (0.821)
		Weighted median	1.737	0.095-31.740	0.710	
		MR-Egger	1.751	0.023-132.749	0.800	
		MR-PRESSO	2.734	0.406-18.387	0.304	

ALP, alkaline phosphatase; ALT, alanine aminotransferase.

## Discussion

We have used data from large GWASs to evaluate the causal relevance of metabolic disorders and gout or serum urate using the MR method. Our analysis showed that genetically predicted fasting insulin, BMI, TG, blood pressure, and ALT were robust associated with gout or serum urate. Further research indicated that HDL and serum urate as well as gout have a bidirectional causal effect on each other.

A meta-analysis of 23 observational studies with 575,284 gout patients showed that the incidence of diabetes in gout population increased as age increased (28). A case–control study indicated that the relative risk for incident gout among diabetes patients, as compared with individuals with no diabetes, was 0.67 (29). Pan et al.’s research came to similar conclusions (30). Using different types of drugs to treat diabetes would affect the risk of gout. A cohort study showed that adults with T2DM prescribed a sodium-glucose cotransporter-2 (SGLT2) inhibitor had a lower rate of gout than those prescribed a glucagon-like peptide-1 (GLP1 agonist) (31). For T2DM, we found no evidence of a causal relationship with serum urate and gout. We speculate that T2DM may affect uric acid metabolism by affecting other metabolic indicators, rather than directly increasing serum urate *via* genetic variants. In addition, we also studied the other glycemic traits, including fasting glucose and fasting insulin. A study conducted in economically developing regions of northwest China found that participants with higher fasting blood glucose had higher levels of serum uric acid (9). Furthermore, urinary uric acid clearance appears to decrease in proportion to increases in insulin resistance in normal volunteers, leading to an increase in serum uric acid concentration (32). Our results confirm that the associations between fasting insulin and serum urate are causal, but not gout. Gout is caused by long-standing hyperuricemia, and although fasting insulin can genetically elevate in serum urate, the concentration or duration of the elevated serum urate is not sufficient to cause gout.

Previous epidemiological studies have suggested that general obesity measured by BMI may be a risk factor for gout and serum urate (33, 34). Zhou et al. reported that once the BMI was higher than 19.12 kg/m^2^ for men or 21.3 kg/m^2^ for women, each 1-kg/m^2^ increase in BMI was related to a 5.10-fold increment for men and a 3.93-fold increment for women in serum urate levels (35). Moreover, a prospective study showed that women with general obesity were more likely to progress from hyperuricemia to gout (11). In the current study, we found a positive association with gout or serum urate for genetically predicted BMI in European-ancestry individuals but not for abdominal adiposity as measured by WHR adjusted for BMI. Our results are consistent with a previous Mendelian analysis which is a trans-ancestry study, while we only studied the European population, suggesting that BMI may be related to gout in different ancestries (36).

In terms of serum lipid, we observed decreased HDL as a causal risk factor of gout and increased serum urate. This is similar to the results reported in observational studies (11, 37). Studies have shown that patients with gout have elevated blood lipid levels, and Mendelian analysis found that high serum urate levels were associated with increased risk of hypercholesterolemia (11, 38). Compared with MR analysis published previously, MR-PRESSO was conducted in our research to evaluate pleiotropy and identify outlier variants based on their observed distance from the regression line and estimate results after correction of outliers. It may exclude the pleiotropy of an instrumental variable to a greater extent. Our analysis showed that HDL and serum urate as well as gout have a bidirectional causal effect on each other good management of one is potentially beneficial to the other. However, in this article we did not discover the relationship of causality between LDL, TC, and serum urate as well as gout.

Also, the causal relationship between blood pressure, liver function, and gout or serum urate was investigated. A prospective cohort study including 6,424 hyperuricemia-free participants proved positive relationships between hypertension and hyperuricemia (39). A meta-analysis including 10 articles showed that hypertensive individuals were more likely to develop gout compared with normotensive individuals (33). In terms of blood pressure, epidemiological studies indicated that patients with hyperuricemia and gout had higher SBP, DBP, and PP (40, 41). In the current study, we found that higher SBP was causally associated with risk of gout or serum urate concentrations. Increased evidence indicates that hyperuricemia and gout are associated with increased prevalence and disease severity of non-alcoholic fatty liver disease (NAFLD), and NAFLD can predict hyperuricemia as well (42). Although there are few studies about the effect of liver dysfunction on gout and serum urate, we found that elevated circulating ALT was related with increased serum urate through MR analysis.

There were also some limitations in our study. Although this article mainly selected the European population as the object for study, the GWAS on lipid included a part of the non-European population. Moreover, we used MR-Egger and MR-PRESSO to control pleiotropy. However, the possibility that pleiotropy may have influenced the results cannot entirely be ruled out as in any Mendelian randomization study. MR analysis assumes a linear relation between each genetic instrument and the risk factor of interest, as well as a log-linear association between the risk factors and outcomes. The estimated effects may not be representative of the effects of the traits in the extremes of their distributions.

## Conclusions

In conclusion, our findings from the genetic study provide support that metabolic factors including BMI, HDL, and SBP are causally associated with serum urate levels and gout risk. Among the above metabolic factors, HDL and serum urate as well as gout have a bidirectional causal effect. In addition, our study demonstrates that variations in fasting insulin, TG, and ALT only have a positive causal effect on serum urate concentrations.

## Data availability statement

The original contributions presented in the study are included in the article/**Supplementary Material**. Further inquiries can be directed to the corresponding author.

## Ethics statement

Ethical review and approval was not required for the study on human participants in accordance with the local legislation and institutional requirements. The patients/participants provided their written informed consent to participate in this study. Written informed consent was obtained from the individual(s) for the publication of any potentially identifiable images or data included in this article.

## Author contributions

YY and WX designed the study. YY, DW, and SH analyzed the data, made the figures, and drafted the manuscript. YL, and HX reviewed and supervised the manuscript. All authors reviewed the manuscript and approved the final version of manuscript.

## Acknowledgments

The authors thank all investigators for sharing these data.

## Conflict of interest

The authors declare that the research was conducted in the absence of any commercial or financial relationships that could be construed as a potential conflict of interest.

## Publisher’s note

All claims expressed in this article are solely those of the authors and do not necessarily represent those of their affiliated organizations, or those of the publisher, the editors and the reviewers. Any product that may be evaluated in this article, or claim that may be made by its manufacturer, is not guaranteed or endorsed by the publisher.

## Supplementary material

Supplementary Figure 1 The relationship between type 2 diabetes mellitus (T2DM)-associated single nucleotide polymorphisms (SNPs) and risk of increased serum urate and gout. Three different methods [inverse variance weighted (IVW) approach, MR-Egger, and weighted median] were used. **(A)** The scattered plot of SNPs associated with T2DM and their risk on increased serum urate. **(B)** The scattered plot of SNPs associated with T2DM and their risk on gout.

Supplementary Figure 2 The relationship between fasting insulin-associated single nucleotide polymorphisms (SNPs) and risk of increased serum urate and gout. Three different methods [inverse variance weighted (IVW) approach, MR-Egger, and weighted median] were used. **(A)** The scattered plot of SNPs associated with fasting insulin and their risk on increased serum urate. **(B)** The scattered plot of SNPs associated with fasting insulin and their risk on gout.

Supplementary Figure 3 The relationship between fasting glucose-associated single nucleotide polymorphisms (SNPs) and risk of increased serum urate and gout. Three different methods [inverse variance weighted (IVW) approach, MR-Egger, and weighted median] were used. **(A)** The scattered plot of SNPs associated with fasting glucose and their risk on increased serum urate. **(B)** The scattered plot of SNPs associated with fasting glucose and their risk on gout.

Supplementary Figure 4 The relationship between body mass index (BMI)-associated single nucleotide polymorphisms (SNPs) and risk of increased serum urate and gout. Three different methods [inverse variance weighted (IVW) approach, MR-Egger, and weighted median] were used. **(A)** The scattered plot of SNPs associated with BMI and their risk on increased serum urate. **(B)** The scattered plot of SNPs associated with BMI and their risk on gout.

Supplementary Figure 5 The relationship between waist-to-hip ratio adjusted for body mass index (WHRadjBM)-associated single nucleotide polymorphisms (SNPs) and risk of increased serum urate and gout. Three different methods [inverse variance weighted (IVW) approach, MR-Egger, and weighted median] were used. **(A)** The scattered plot of SNPs associated with WHRadjBM and their risk on increased serum urate. **(B)** The scattered plot of SNPs associated with WHRadjBM and their risk on gout.

Supplementary Figure 6 The relationship between high-density lipoprotein cholesterol (HDL)-associated single nucleotide polymorphisms (SNPs) and risk of increased serum urate and gout. Three different methods [inverse variance weighted (IVW) approach, MR-Egger, and weighted median] were used. **(A)** The scattered plot of SNPs associated with HDL and their risk on increased serum urate. **(B)** The scattered plot of SNPs associated with HDL and their risk on gout.

Supplementary Figure 7 The relationship between triglycerides (TG)-associated single nucleotide polymorphisms (SNPs) and risk of increased serum urate and gout. Three different methods [inverse variance weighted (IVW) approach, MR-Egger, and weighted median] were used. **(A)** The scattered plot of SNPs associated with TG and their risk on increased serum urate. **(B)** The scattered plot of SNPs associated with TG and their risk on gout.

Supplementary Figure 8 The relationship between low-density lipoprotein cholesterol (LDL)-associated single nucleotide polymorphisms (SNPs) and risk of increased serum urate and gout. Three different methods [inverse variance weighted (IVW) approach, MR-Egger, and weighted median] were used. **(A)** The scattered plot of SNPs associated with LDL and their risk on increased serum urate. **(B)** The scattered plot of SNPs associated with LDL and their risk on gout.

Supplementary Figure 9 The relationship between total cholesterol (TC)-associated single nucleotide polymorphisms (SNPs) and risk of increased serum urate and gout. Three different methods [inverse variance weighted (IVW) approach, MR-Egger, and weighted median] were used. **(A)** The scattered plot of SNPs associated with TC and their risk on increased serum urate. **(B)** The scattered plot of SNPs associated with TC and their risk on gout.

Supplementary Figure 10 The relationship between systolic blood pressure (SBP)-associated single nucleotide polymorphisms (SNPs) and risk of increased serum urate and gout. Three different methods [inverse variance weighted (IVW) approach, MR-Egger, and weighted median] were used. **(A)** The scattered plot of SNPs associated with SBP and their risk on increased serum urate. **(B)** The scattered plot of SNPs associated with SBP and their risk on gout.

Supplementary Figure 11 The relationship between pulse pressure (PP)-associated single nucleotide polymorphisms (SNPs) and risk of increased serum urate and gout. Three different methods [inverse variance weighted (IVW) approach, MR-Egger, and weighted median] were used. **(A)** The scattered plot of SNPs associated with PP and their risk on increased serum urate. **(B)** The scattered plot of SNPs associated with PP and their risk on gout.

Supplementary Figure 12 The relationship between diastolic blood pressure (DBP)-associated single nucleotide polymorphisms (SNPs) and risk of increased serum urate and gout. Three different methods [inverse variance weighted (IVW) approach, MR-Egger, and weighted median] were used. **(A)** The scattered plot of SNPs associated with DBP and their risk on increased serum urate. **(B)** The scattered plot of SNPs associated with DBP and their risk on gout.

Supplementary Figure 13 The relationship between alanine aminotransferase (ALT)-associated single nucleotide polymorphisms (SNPs) and risk of increased serum urate and gout. Three different methods [inverse variance weighted (IVW) approach, MR-Egger, and weighted median] were used. **(A)** The scattered plot of SNPs associated with ALT and their risk on increased serum urate. **(B)** The scattered plot of SNPs associated with ALT and their risk on gout.

Supplementary Figure 14 The relationship between alkaline phosphatase (ALP)-associated single nucleotide polymorphisms (SNPs) and risk of increased serum urate and gout. Three different methods [inverse variance weighted (IVW) approach, MR-Egger, and weighted median] were used. **(A)** The scattered plot of SNPs associated with ALP and their risk on increased serum urate. **(B)** The scattered plot of SNPs associated with ALP and their risk on gout.
