# Supplementary figures and images for: The role of obesity, type 2 diabetes, and metabolic factors in gout: A Mendelian randomization study
Source: Front Endocrinol (Lausanne). 2022 Aug 5;13:917056. doi: 10.3389/fendo.2022.917056 (PMC9388832; doi:10.3389/fendo.2022.917056)

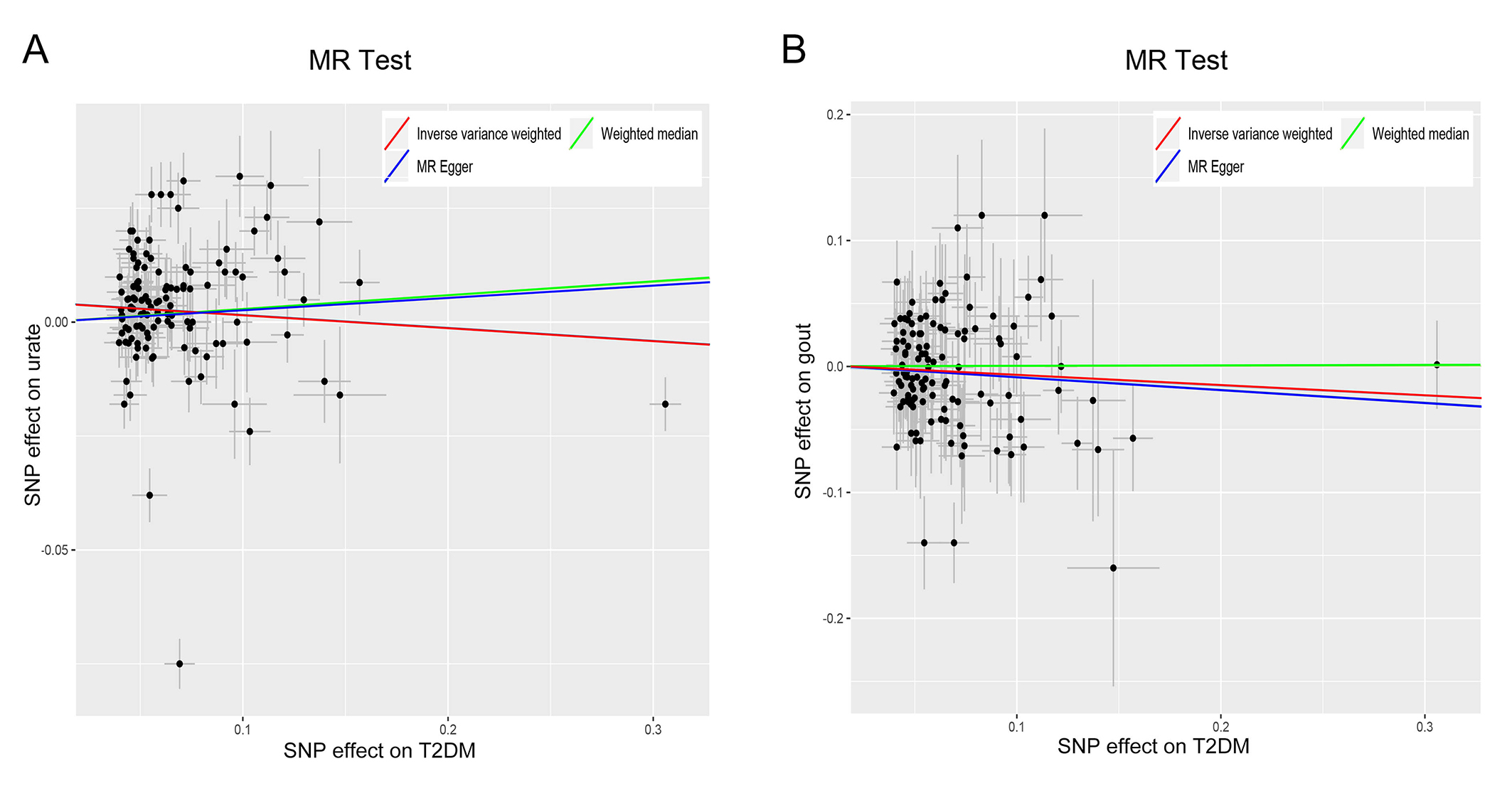

Supplement: Supplementary Figure 1 — The relationship between type 2 diabetes mellitus (T2DM)-associated single nucleotide polymorphisms (SNPs) and risk of increased serum urate and gout. Three different methods [inverse variance weighted (IVW) approach, MR-Egger, and weighted median] were used. (A) The scattered plot of SNPs associated with T2DM and their risk on increased serum urate. (B) The scattered plot of SNPs associated with T2DM and their risk on gout. [file Image_1.jpeg]

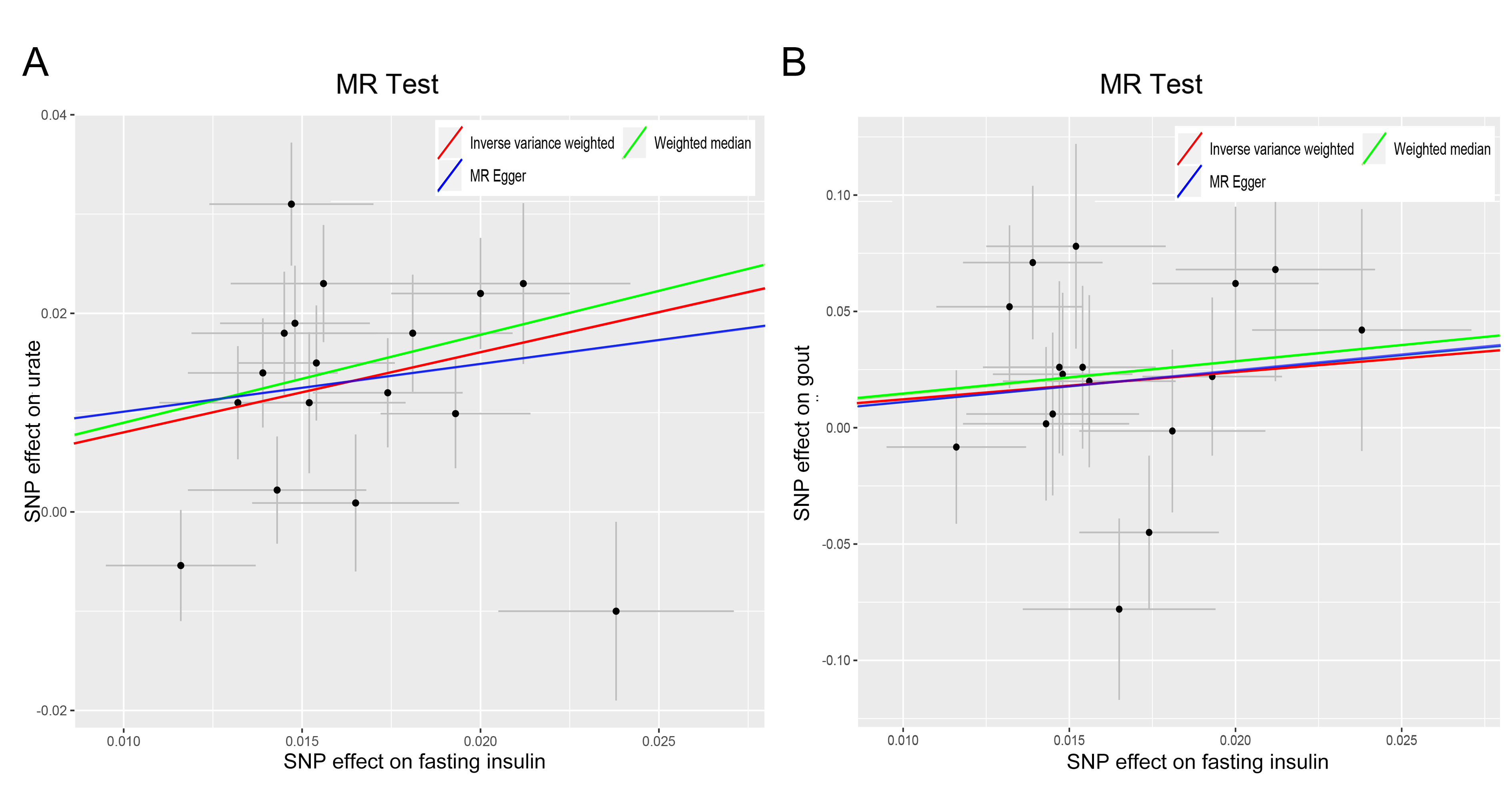

Supplement: Supplementary Figure 2 — The relationship between fasting insulin-associated single nucleotide polymorphisms (SNPs) and risk of increased serum urate and gout. Three different methods [inverse variance weighted (IVW) approach, MR-Egger, and weighted median] were used. (A) The scattered plot of SNPs associated with fasting insulin and their risk on increased serum urate. (B) The scattered plot of SNPs associated with fasting insulin and their risk on gout. [file Image_2.jpeg]

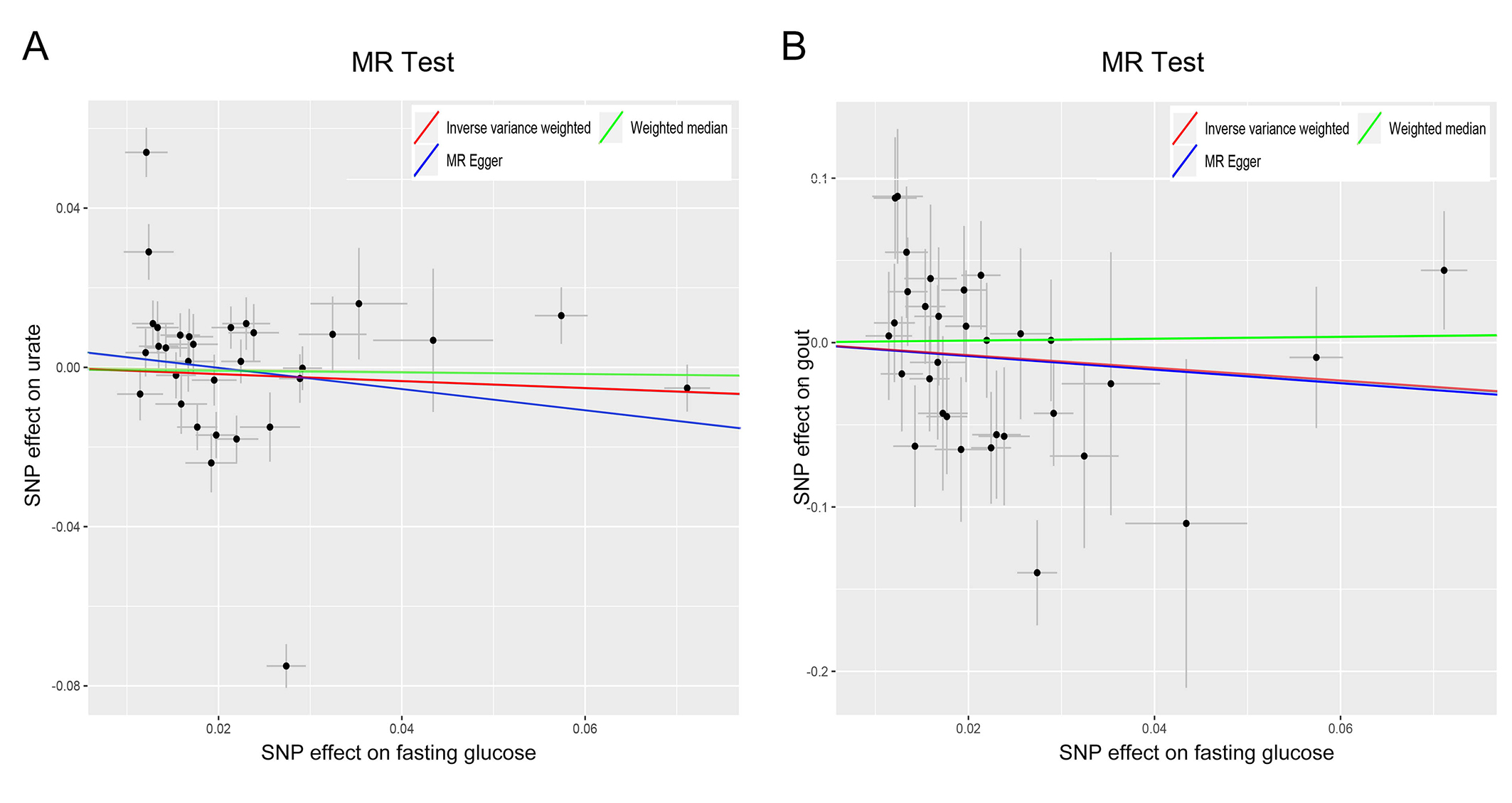

Supplement: Supplementary Figure 3 — The relationship between fasting glucose-associated single nucleotide polymorphisms (SNPs) and risk of increased serum urate and gout. Three different methods [inverse variance weighted (IVW) approach, MR-Egger, and weighted median] were used. (A) The scattered plot of SNPs associated with fasting glucose and their risk on increased serum urate. (B) The scattered plot of SNPs associated with fasting glucose and their risk on gout. [file Image_3.jpeg]

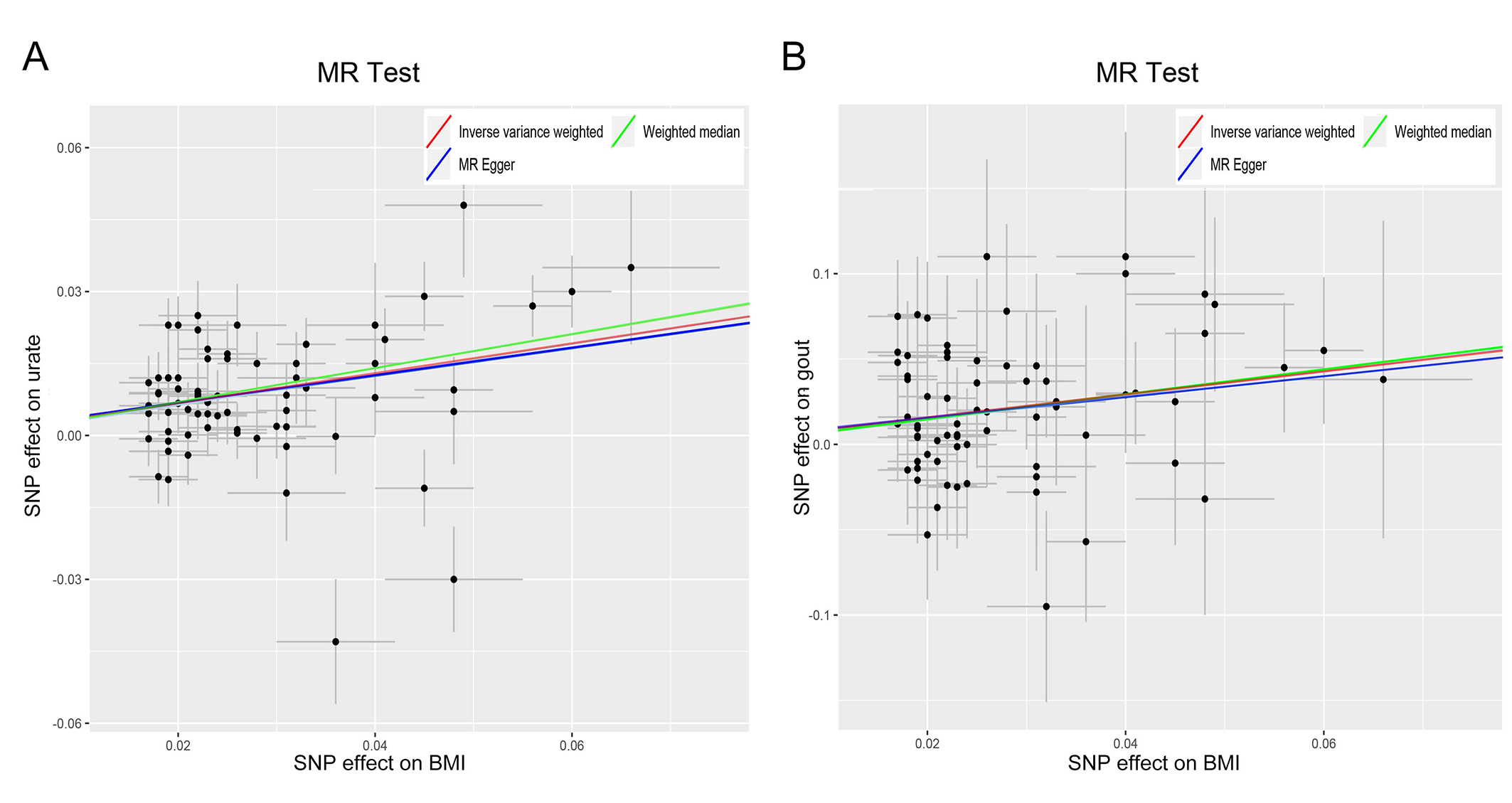

Supplement: Supplementary Figure 4 — The relationship between body mass index (BMI)-associated single nucleotide polymorphisms (SNPs) and risk of increased serum urate and gout. Three different methods [inverse variance weighted (IVW) approach, MR-Egger, and weighted median] were used. (A) The scattered plot of SNPs associated with BMI and their risk on increased serum urate. (B) The scattered plot of SNPs associated with BMI and their risk on gout. [file Image_4.jpeg]

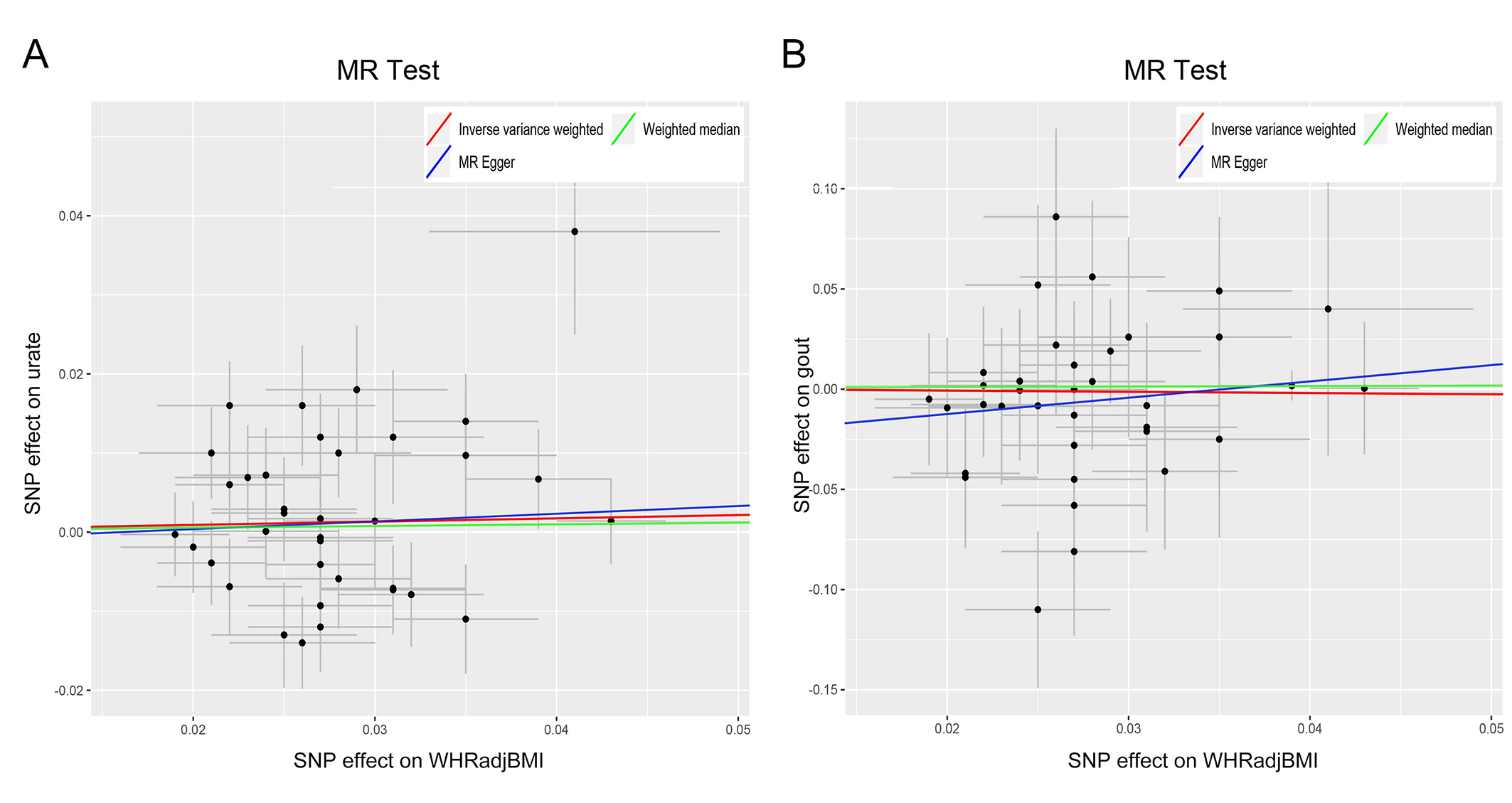

Supplement: Supplementary Figure 5 — The relationship between waist-to-hip ratio adjusted for body mass index (WHRadjBM)-associated single nucleotide polymorphisms (SNPs) and risk of increased serum urate and gout. Three different methods [inverse variance weighted (IVW) approach, MR-Egger, and weighted median] were used. (A) The scattered plot of SNPs associated with WHRadjBM and their risk on increased serum urate. (B) The scattered plot of SNPs associated with WHRadjBM and their risk on gout. [file Image_5.jpeg]

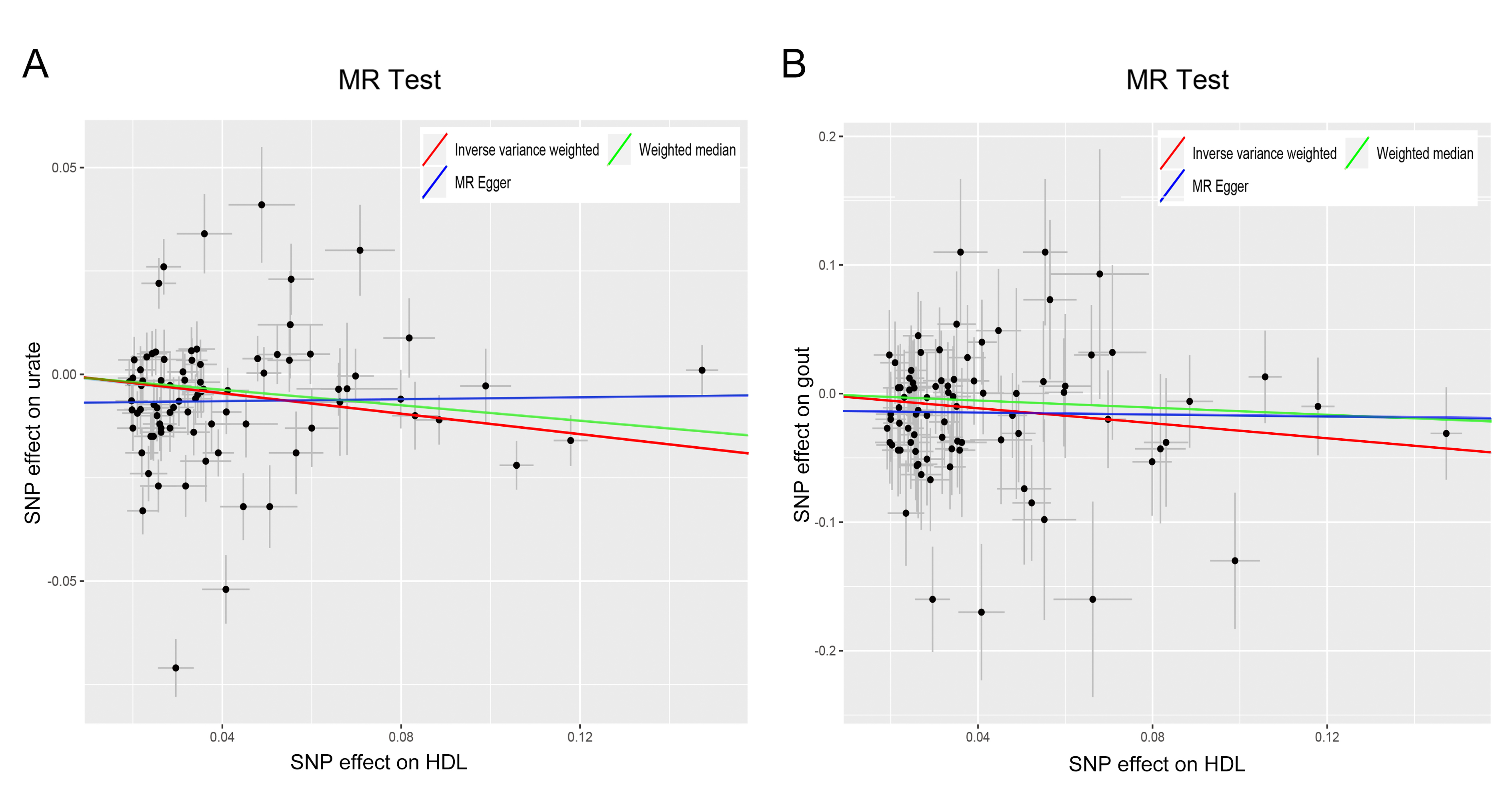

Supplement: Supplementary Figure 6 — The relationship between high-density lipoprotein cholesterol (HDL)-associated single nucleotide polymorphisms (SNPs) and risk of increased serum urate and gout. Three different methods [inverse variance weighted (IVW) approach, MR-Egger, and weighted median] were used. (A) The scattered plot of SNPs associated with HDL and their risk on increased serum urate. (B) The scattered plot of SNPs associated with HDL and their risk on gout. [file Image_6.jpeg]

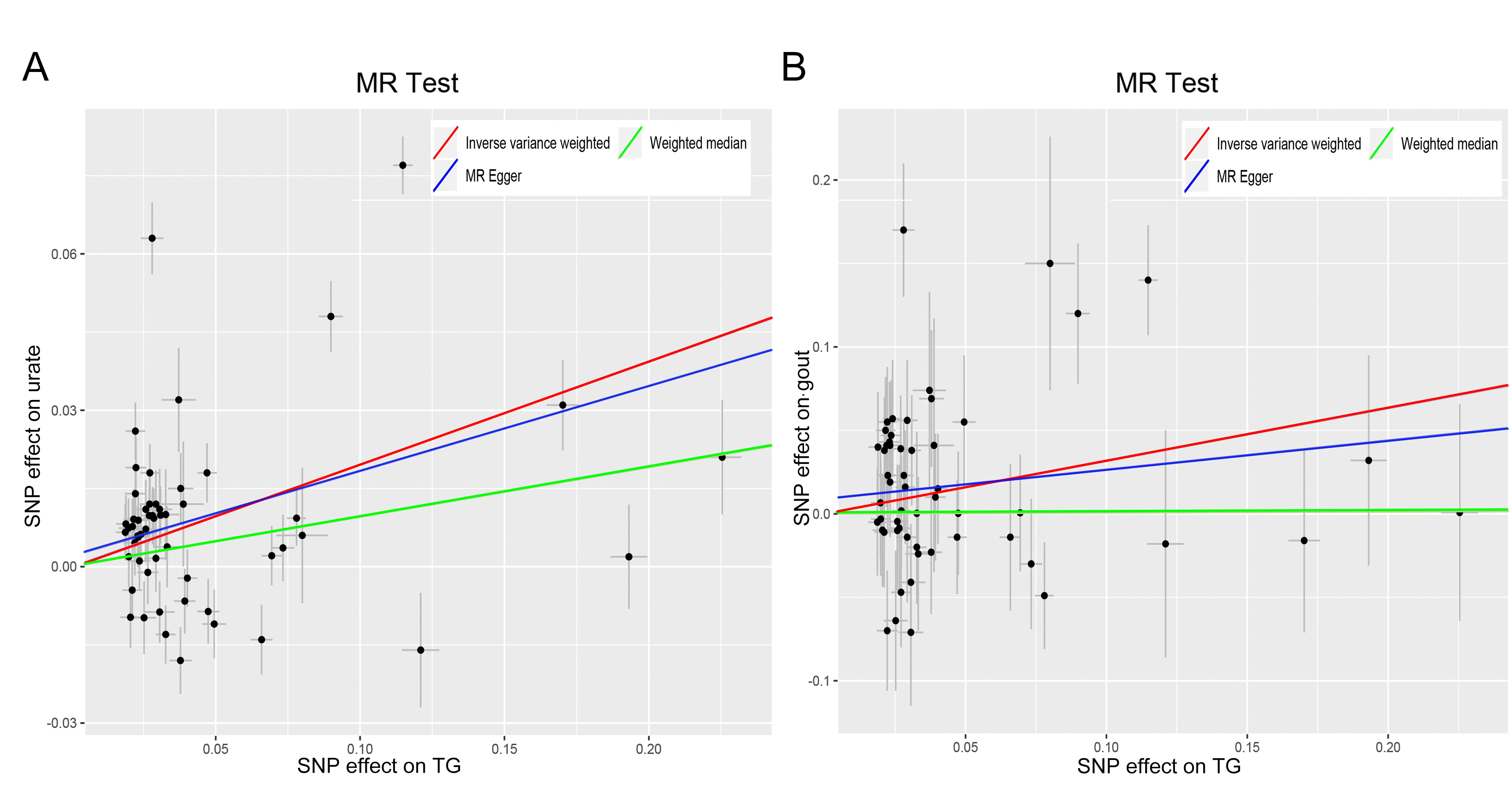

Supplement: Supplementary Figure 7 — The relationship between triglycerides (TG)-associated single nucleotide polymorphisms (SNPs) and risk of increased serum urate and gout. Three different methods [inverse variance weighted (IVW) approach, MR-Egger, and weighted median] were used. (A) The scattered plot of SNPs associated with TG and their risk on increased serum urate. (B) The scattered plot of SNPs associated with TG and their risk on gout. [file Image_7.jpeg]

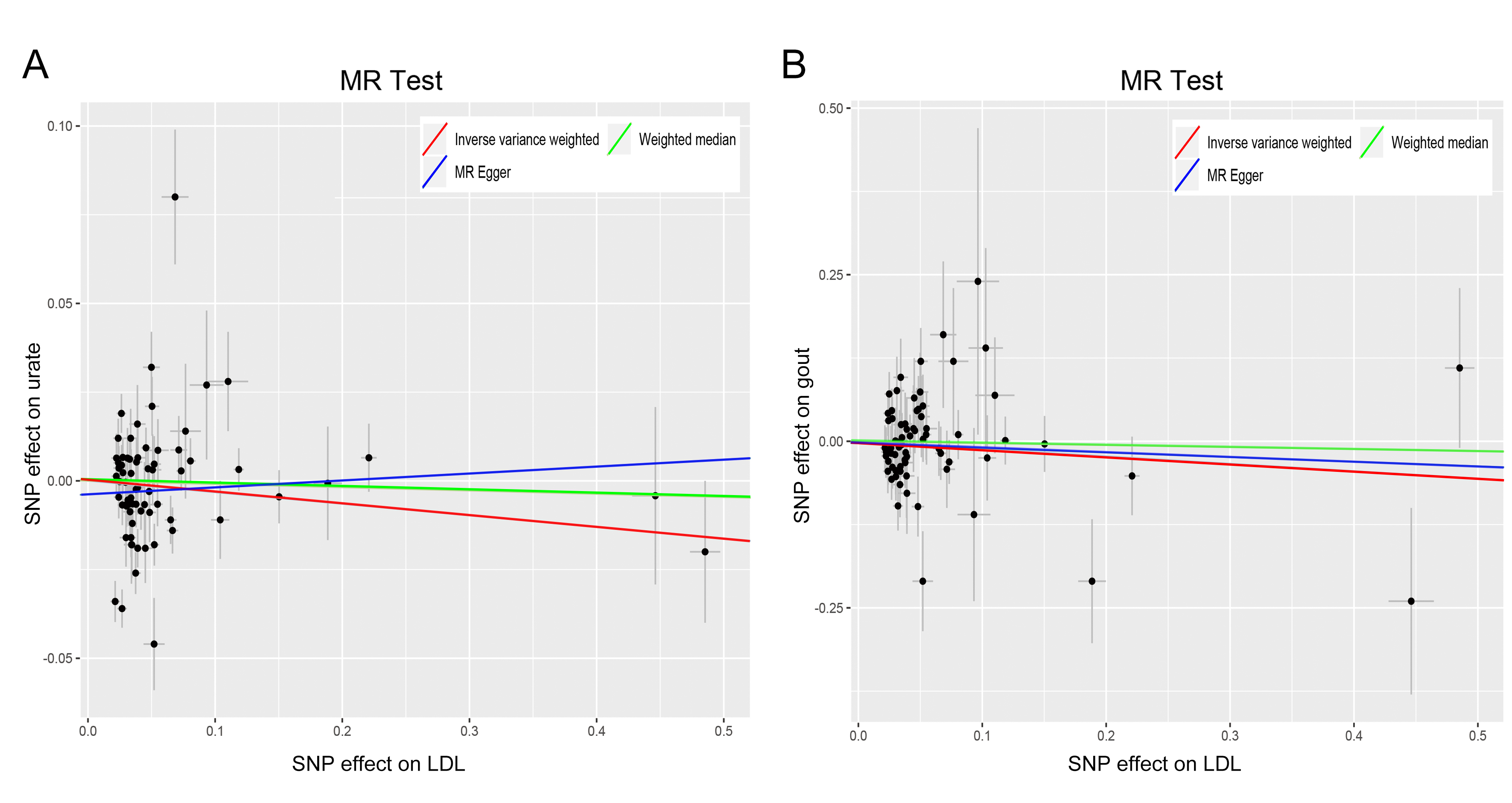

Supplement: Supplementary Figure 8 — The relationship between low-density lipoprotein cholesterol (LDL)-associated single nucleotide polymorphisms (SNPs) and risk of increased serum urate and gout. Three different methods [inverse variance weighted (IVW) approach, MR-Egger, and weighted median] were used. (A) The scattered plot of SNPs associated with LDL and their risk on increased serum urate. (B) The scattered plot of SNPs associated with LDL and their risk on gout. [file Image_8.jpeg]

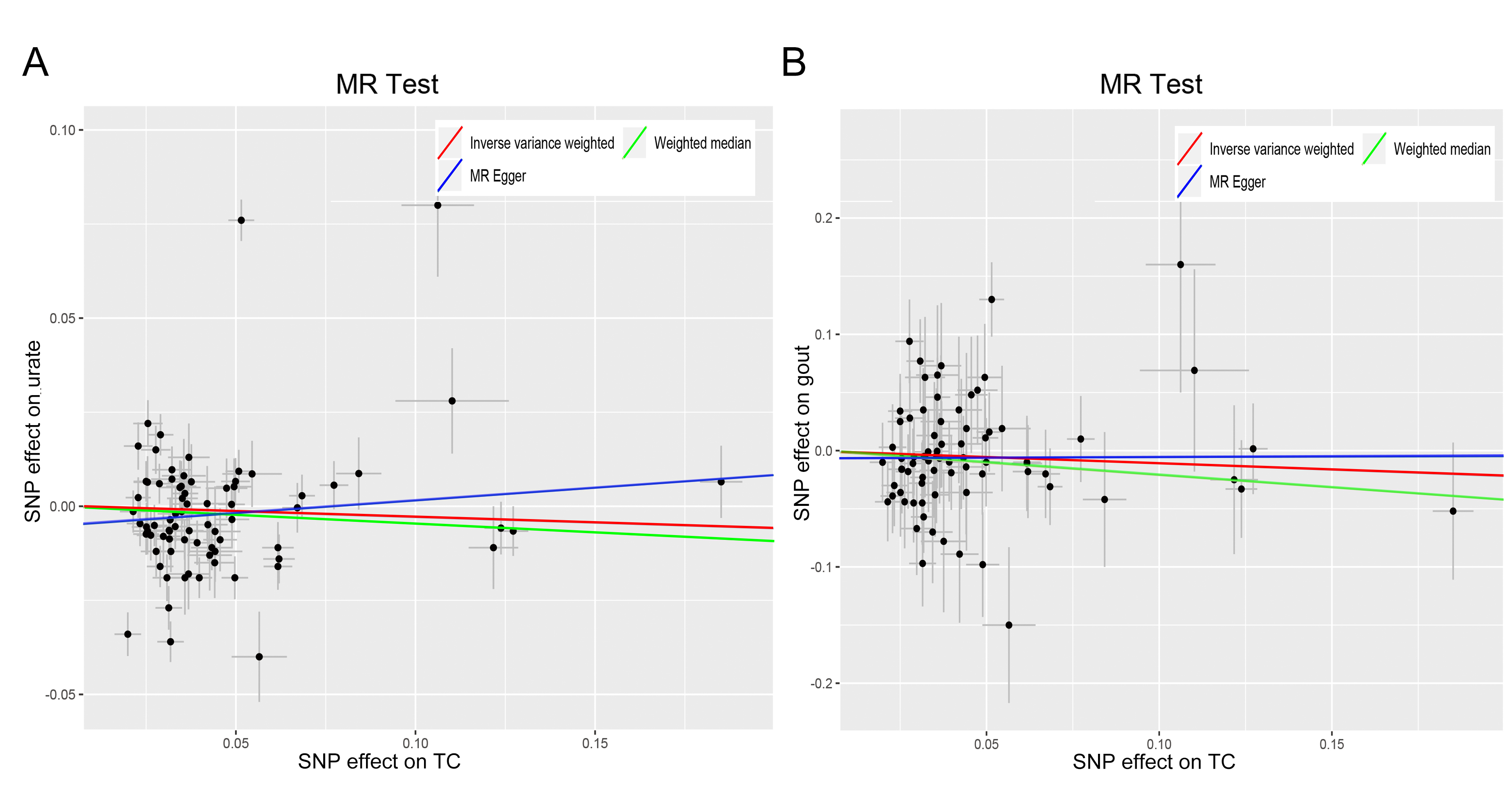

Supplement: Supplementary Figure 9 — The relationship between total cholesterol (TC)-associated single nucleotide polymorphisms (SNPs) and risk of increased serum urate and gout. Three different methods [inverse variance weighted (IVW) approach, MR-Egger, and weighted median] were used. (A) The scattered plot of SNPs associated with TC and their risk on increased serum urate. (B) The scattered plot of SNPs associated with TC and their risk on gout. [file Image_9.jpeg]

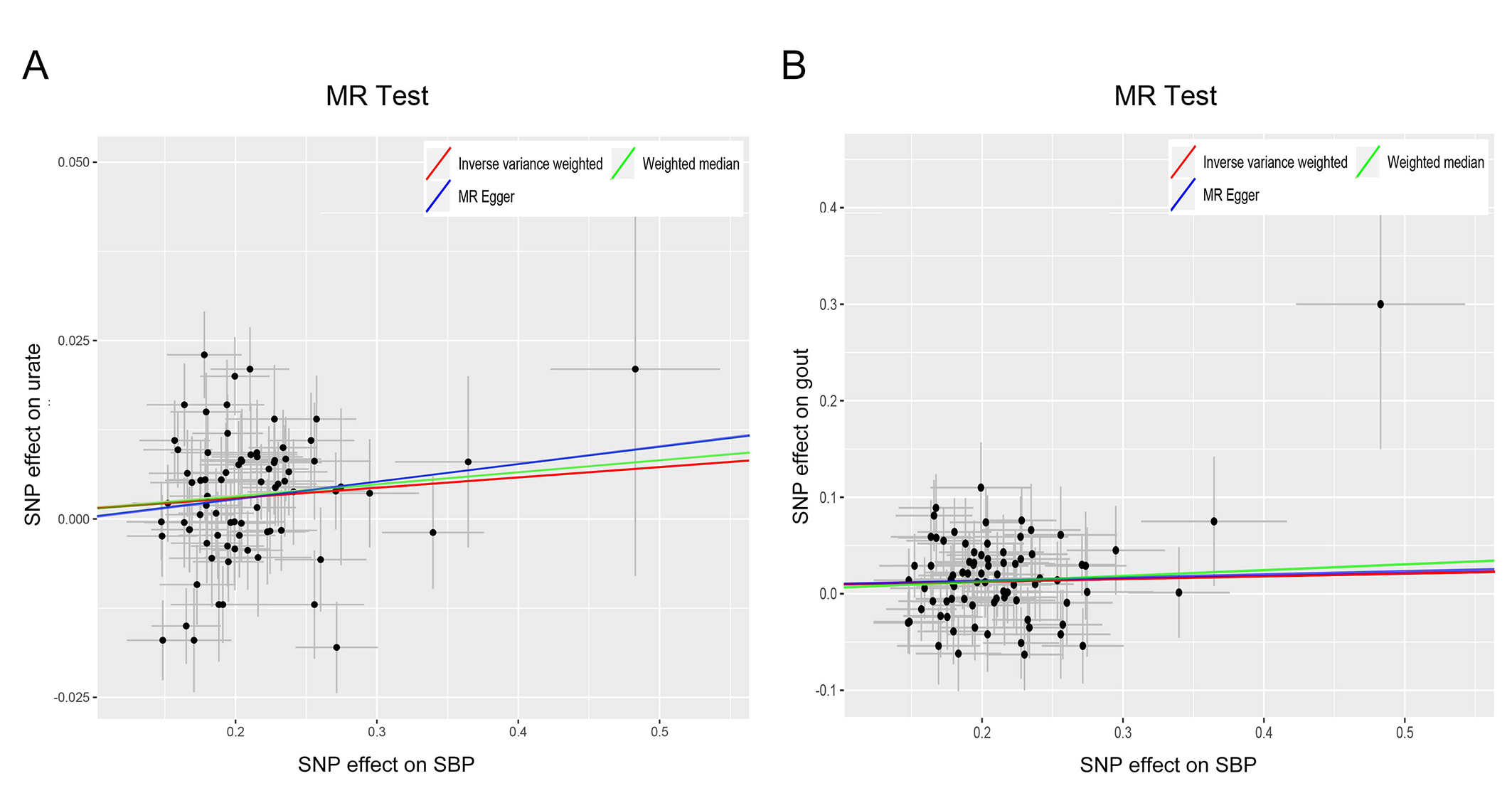

Supplement: Supplementary Figure 10 — The relationship between systolic blood pressure (SBP)-associated single nucleotide polymorphisms (SNPs) and risk of increased serum urate and gout. Three different methods [inverse variance weighted (IVW) approach, MR-Egger, and weighted median] were used. (A) The scattered plot of SNPs associated with SBP and their risk on increased serum urate. (B) The scattered plot of SNPs associated with SBP and their risk on gout. [file Image_10.jpeg]

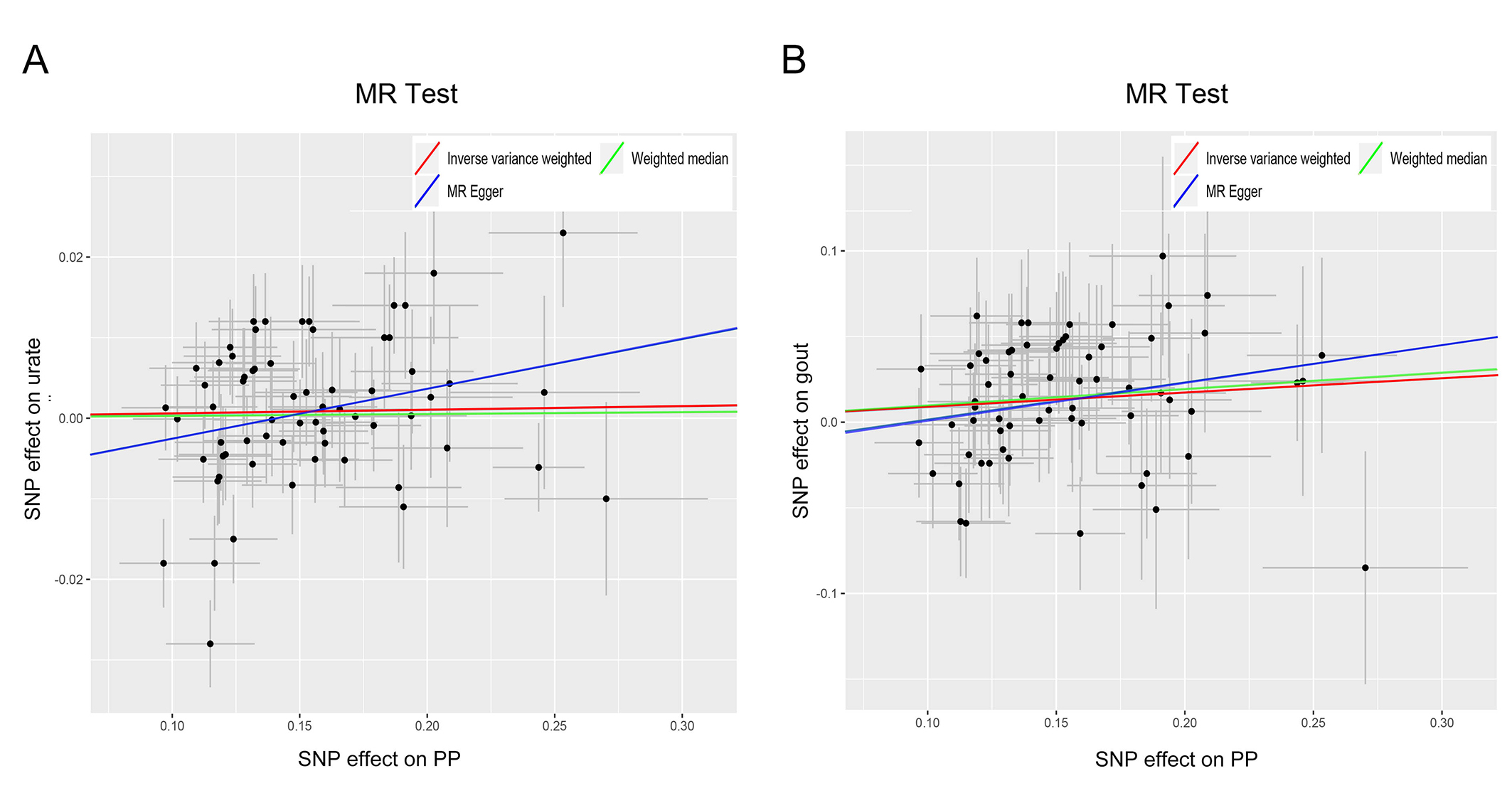

Supplement: Supplementary Figure 11 — The relationship between pulse pressure (PP)-associated single nucleotide polymorphisms (SNPs) and risk of increased serum urate and gout. Three different methods [inverse variance weighted (IVW) approach, MR-Egger, and weighted median] were used. (A) The scattered plot of SNPs associated with PP and their risk on increased serum urate. (B) The scattered plot of SNPs associated with PP and their risk on gout. [file Image_11.jpeg]

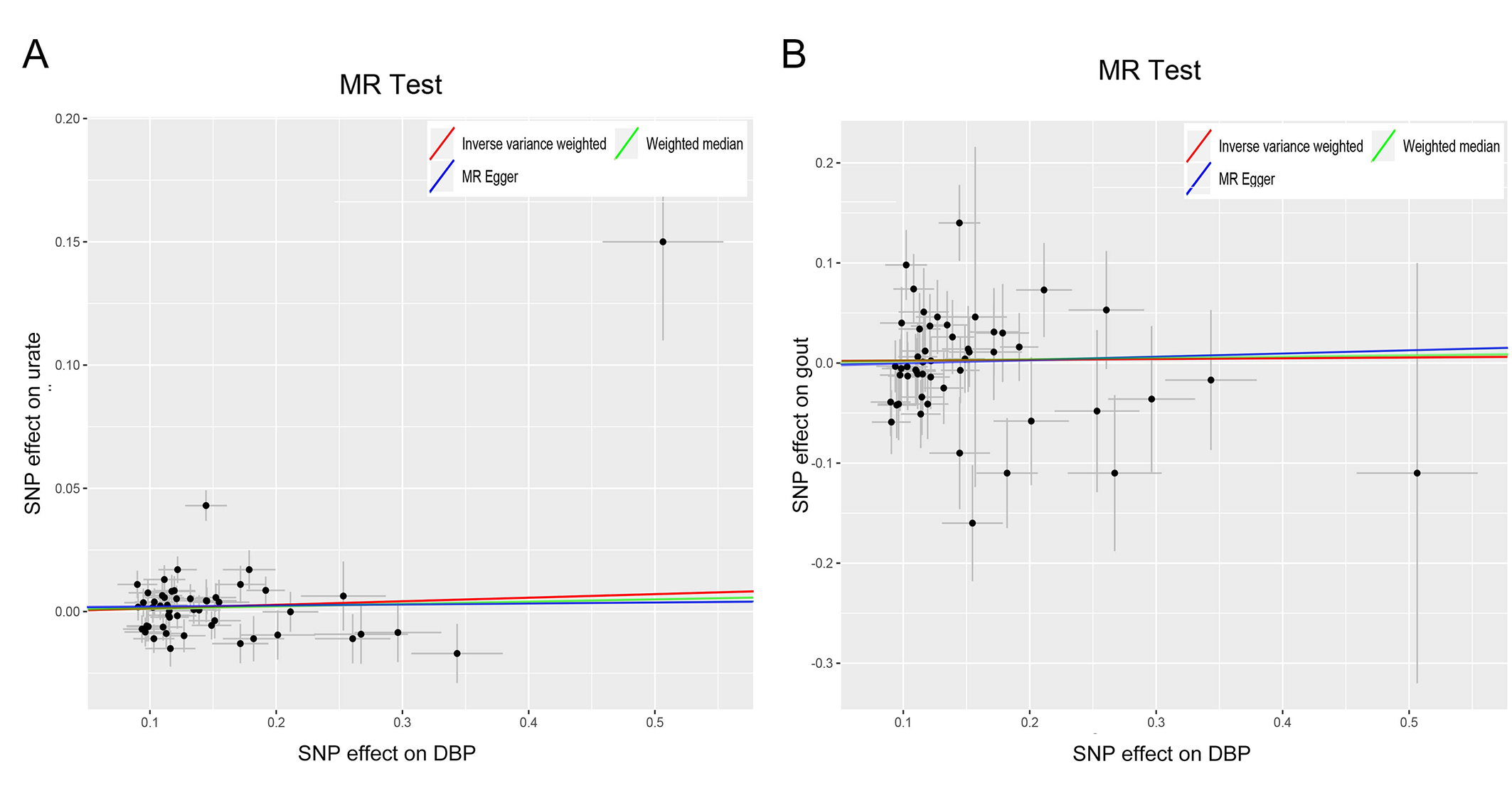

Supplement: Supplementary Figure 12 — The relationship between diastolic blood pressure (DBP)-associated single nucleotide polymorphisms (SNPs) and risk of increased serum urate and gout. Three different methods [inverse variance weighted (IVW) approach, MR-Egger, and weighted median] were used. (A) The scattered plot of SNPs associated with DBP and their risk on increased serum urate. (B) The scattered plot of SNPs associated with DBP and their risk on gout. [file Image_12.jpeg]

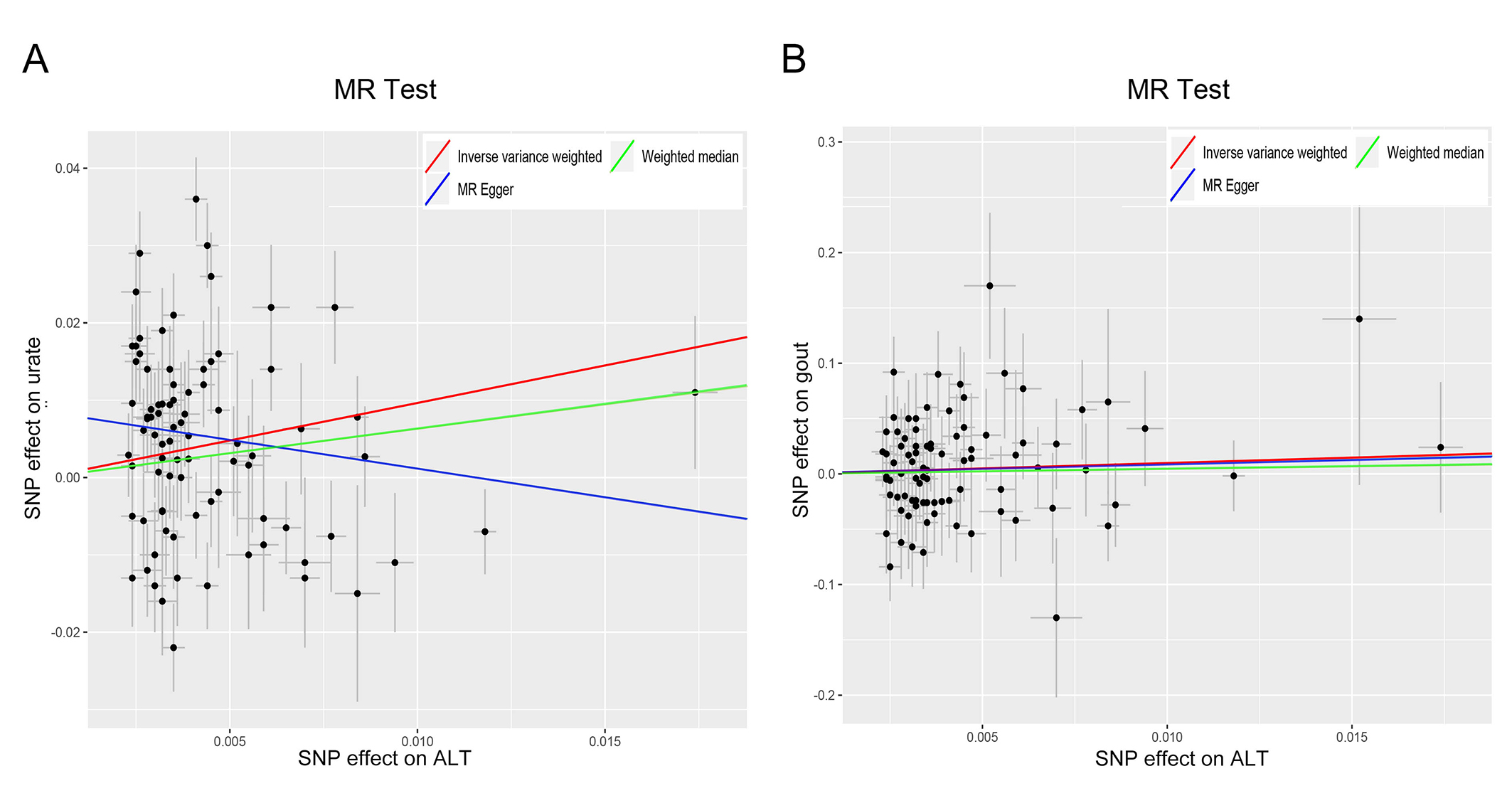

Supplement: Supplementary Figure 13 — The relationship between alanine aminotransferase (ALT)-associated single nucleotide polymorphisms (SNPs) and risk of increased serum urate and gout. Three different methods [inverse variance weighted (IVW) approach, MR-Egger, and weighted median] were used. (A) The scattered plot of SNPs associated with ALT and their risk on increased serum urate. (B) The scattered plot of SNPs associated with ALT and their risk on gout. [file Image_13.jpeg]

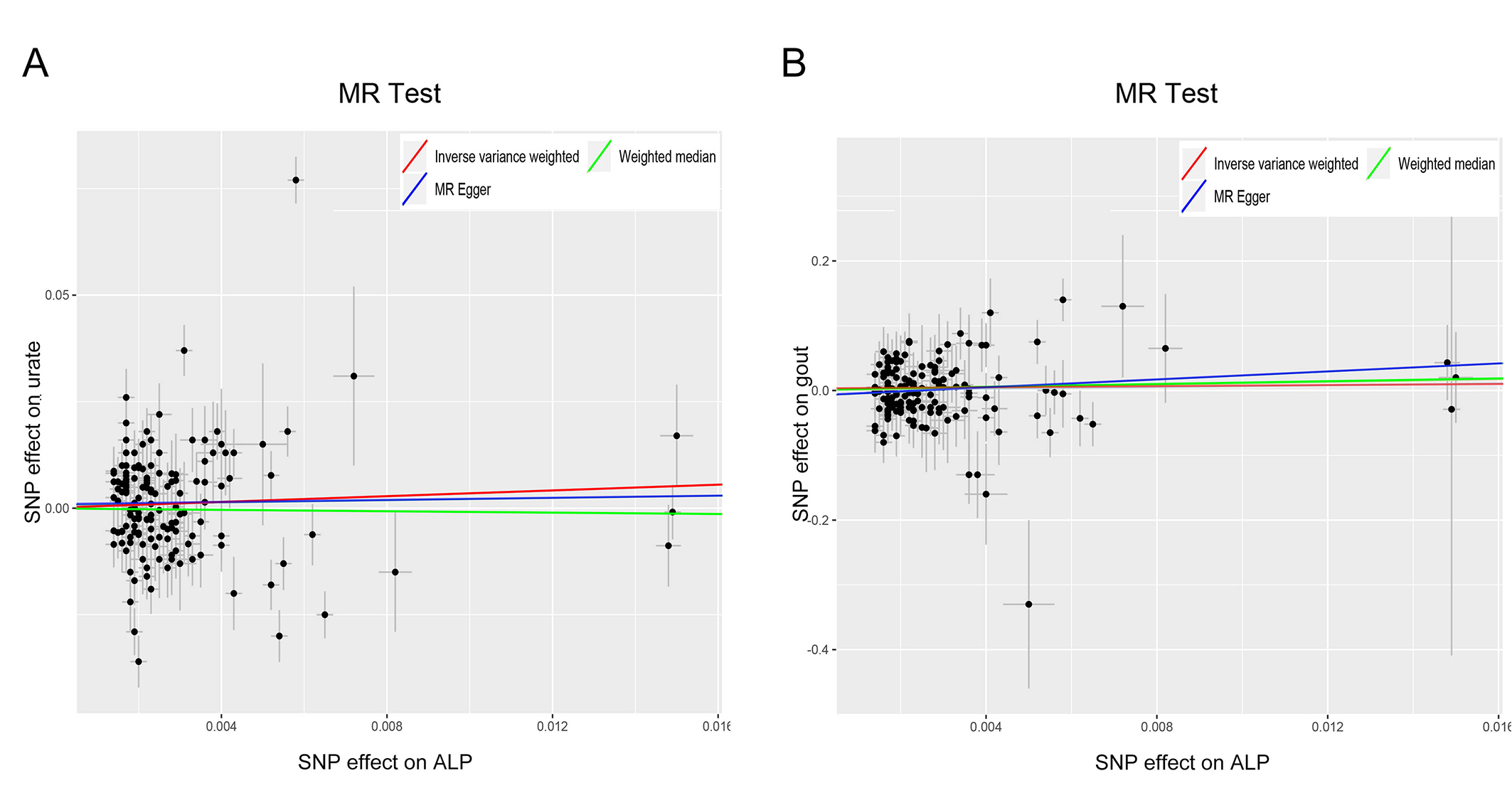

Supplement: Supplementary Figure 14 — The relationship between alkaline phosphatase (ALP)-associated single nucleotide polymorphisms (SNPs) and risk of increased serum urate and gout. Three different methods [inverse variance weighted (IVW) approach, MR-Egger, and weighted median] were used. (A) The scattered plot of SNPs associated with ALP and their risk on increased serum urate. (B) The scattered plot of SNPs associated with ALP and their risk on gout. [file Image_14.jpeg]
